# Preparation of 2-Aminothiazole-Functionalized Poly(glycidyl methacrylate) Microspheres and Their Excellent Gold Ion Adsorption Properties

**DOI:** 10.3390/polym10020159

**Published:** 2018-02-08

**Authors:** Chao Xiong, Shixing Wang, Libo Zhang, Ying Li, Yang Zhou, Jinhui Peng

**Affiliations:** 1 Faculty of Metallurgical and Energy Engineering, Kunming University of Science and Technology, Kunming 650093, China; m15687371837@163.com (C.X.); zhanglibopaper@126.com (L.Z.); liyingkmust@126.com (Y.L.); jhpeng@kmust.edu.cn (J.P.); 2 State Key Laboratory of Complex Nonferrous Metal Resources Clean Utilization, Kunming University of Science and Technology, Kunming 650093, China; 3 School of Textile Science and Engineering, Wuhan Textile University, Wuhan 430200, China; zhouyangnano@163.com

**Keywords:** adsorption, gold, reusability, mechanism, selectivity

## Abstract

A new adsorbent(A-PGMA) has been synthesized via functionalizing poly(glycidyl methacrylate) microsphere with 2-aminothiazole and used to adsorb gold ions from aqueous solutions. The adsorbent was characterized by X-ray photoelectron spectroscopy (XPS), Brunauer–Emmett–Teller (BET), Zeta potential, scanning electron microscope (SEM) and Fourier transform infrared spectroscopy (FT-IR). The influence factors such as the pH value of the solution, the initial gold ion concentration and the contact time were examined. Simultaneously, the adsorption process of the gold ion on A-PGMA fitted well with the Langmuir and pseudo-second-order models, respectively. The results showed that the maximum adsorption capacity was 440.54 mg/g and the equilibrium time of adsorption was about 3 h under pH 4. Moreover, the adsorbent has a high reusability after five cycles and good selectivity from coexisting ions, including Zn(II), Mg(II), Cu(II), Ge(IV) and B(III). The adsorption mechanisms of gold ions were ion exchange and chelation between the sulfur and nitrogen groups on the surface of A-PGMA and AuCl_4_^−^. Therefore, the adsorbent has a great potential for adsorption of gold ions from aqueous solutions.

## 1. Introduction

Gold has been known for centuries as a precious metal for its wide applications and outstanding physical and chemical properties, such as excellent ductility and conductivity, high catalytic activity and so on [1,2,3,4]. Therefore, gold is widely used in electronics industry, catalysts, aerospace equipment, medicine and so on [5,6]. In addition, gold has the attributes of money and commodities and is the most effective international reserve for governments and central banks. Because of the limited amount of gold resources in nature, nowadays, the recycling of gold from electronics, spent catalyst wastes and liquid wastes have been taken into consideration by the public and government [7,8].

In recent years, many methods, including membrane filtration [9], ion-exchange [10], precipitation [11], electro dialysis [12], solvent extraction [13] and adsorption methods [14,15,16] have been used to separate and enrich gold ions. The adsorption method is very promising for its high efficiency, simplicity, low amount of secondary pollution and cost-effectiveness [17,18]. At present, many adsorbents have been developed for the adsorption of gold, for example, chitosan, activated carbon and cellulose [19,20,21]. However, the low surface areas/site ratio, low adsorption capacity, low reusable rate, high cost and weak selectivity restrain the actual applicability in gold adsorption. For this perspective, it is very important to develop a novel adsorbent with high capacity and selectivity.

Polymer microspheres have been widely applied into the adsorption of metal ions [22]. Particularly, a resin microsphere has attracted much attention due to its high dispersibility in solvents and large surface area [23,24]. Poly(glycidyl methacrylate) (PGMA) is easy to modify because it consists of epoxy side groups. The epoxy groups can be easily converted into alcohol, amine and acid functional groups [25,26]. PGMA has excellent acid and alkali resistance. In addition, PGMA with various functional groups has good adsorption properties of metal ions. Moreover, the groups containing S and N donor atoms can strongly interact with precious metals based on the theory of hard soft acid-base (HSAB). Therefore, 2-aminothiazole was chosen to chelate with gold according to HSAB because it contains S and N donor atoms.

In this article, a new adsorbent (A-PGMA) has been synthesized via functionalizing poly(glycidyl methacrylate) microsphere with 2-aminothiazole and used to adsorb gold ions from aqueous solutions. A-PGMA was characterized by SEM (scanning electron microscope), BET (Brunauer–Emmett–Teller), Zeta potential, XPS (X-ray photoelectron spectroscopy) and FT-IR (Fourier transform infrared spectroscopy). Meanwhile, the effect factors including pH value of solution, initial gold concentration and contact time were investigated. In addition, the selectivity, reusability, kinetics, isotherms and mechanism were also studied.

## 2. Experimental

### 2.1. Materials

2-Aminothiazole, polyvinylpyrrolidone (PVP), glycidyl methacrylate (GMA), and azobisisobutyronitrile (ABIN) were purchased from Aladdin Chemistry Co. Ltd. (Shanghai, China) Ethanol and acetic acid came from Sinopharm Group Chemical Reagent Co. Ltd. (Shanghai, China). Gold standard solution was purchased from National Nonferrous Metals Research Institute (Beijing, China). A multielement standard solution with a concentration of 100 mg/L (National standard material center) was used to prepare the calibration standard solutions for quantitative analysis. In addition, the pH value of standard solutions was adjusted by HCl solution (0.1 mol/L) and NaOH solution (0.1 mol/L).

### 2.2. Synthesis of A-PGMA Adsorbent

**Scheme 1:**
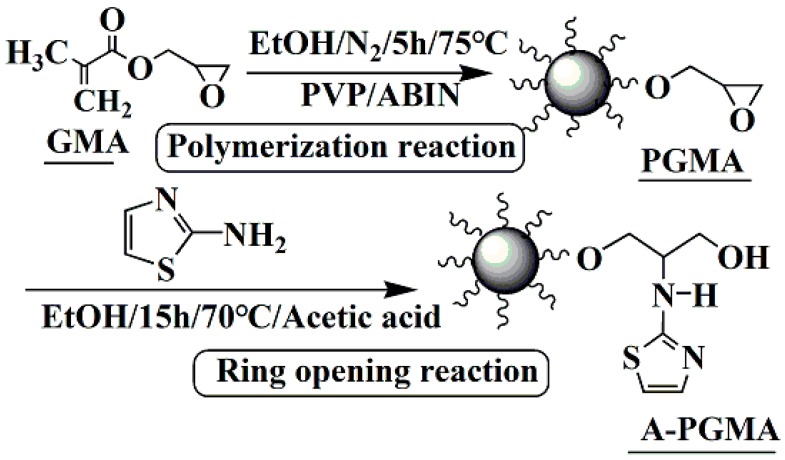
Synthesis process of A-PGMA adsorbent.

Scheme 1 showed the synthesis process of A-PGMA. The first step is to prepare poly(glycidyl methacrylate) microsphere according to the literature [27]. GMA (10 g), ABIN (0.12 g) and PVP (3 g) were added to a 250 mL three-necked flask and thoroughly mixed with 150 mL of ethanol. The mixture was reacted at 70 °C for 5 h under nitrogen protection. Then, the sample was separated by centrifugation, washed five times by ethanol and dried at 50 °C for 12 h and defined as PGMA. The second step is to functionalize poly(glycidyl methacrylate) microspheres with 2-aminothiazole. PGMA (5 g) and 2-aminothiazole (5 g) were added into the mixed solution of ethanol (100 mL) and acetic acid (2 mL) in 250 mL three-necked flask. The temperature was controlled at 70 °C. After 15 h, the suspension was separated by centrifugation. Then, the solid was washed by hot deionized water five times, dried at 50 °C for 12 h under vacuum and defined as A-PGMA. The weight of A-PGMA is 6.8 g.

### 2.3. Adsorption Experiments

In order to study the influence of pH, original concentration of gold ion, contact time and coexisting ions on gold adsorption, all the experiments were implemented by shaking 10 mg of A-PGMA with 20 mL gold solution in the thermostat steam bath vibrator (ZD-85, Allied instrument institute, Jintan, China) at shaking speed of 300 rpm and temperature of 30 °C. Then, the supernatant of each batch experiments were separated and the concentration of gold ions in the solution was detected by inductively coupled plasma optical emission spectrometer. 25 mg A-PGMA was added into 50 mL gold ions solution for the reusability experiments. The adsorption pH was changed in the range of 0.5–7.0 and adsorption kinetics experiments were conducted at 5–600 min. The initial gold ion concentration of 200–700 mg/L was used to examine adsorption isotherms. The selectivity experiments from Zn^2+^, Mg^2+^, Cu^2+^, Ge^4+^ and B were investigated. The equilibrium adsorption capacity (*q*) and removal rate (*R*) of gold ions were calculated as follows Equations (1) and (2):

(1) $$q=\frac{C_{0}-C_{e}}{m}V$$

(2) $$R=\frac{C_{0}-C_{e}}{C_{0}}\times 100\%$$

where *C*_0_ (mg/L) represents the original concentration of gold ions and *C_e_* (mg/L) represents the final concentration of gold ions. *V* (mL) is the volume of auric chloride acid solution and *m* (mg) is the mass of A-PGMA. 

### 2.4. Characterization

The SEM-EDS (scanning electron microscopy) was obtained from Netherlands (Phenom ProX, Royal Dutch Philips Electronics Ltd., Amsterdam, The Netherlands). XPS (X-ray photoelectron spectroscopy) was equipped with a 1486.6 eV radiation source of monochromatized Al K-alpha (Thermo Scientific Co., Waltham, MA, USA). FT-IR (Fourier transform infrared spectroscopy) was analyzed by the Nicolet iS 60 (Thermo Scientific Co., Waltham, M, USA) with a resolution of 4 cm^−1^ and KBr pellets in the range from 400 to 4000 cm^−1^. Zeta potential of the adsorbent was measured by Nano Brook Omni multi angle particle size and high sensitivity Zeta potential analyzer (Brookhaven Instruments Co., Austin, TX, USA). BET surface and pore analyzer (Micromeritics, ASAP 2020, Micromeritics Instrument Corp., Norcross, GA, USA) was employed to test the BET surface area of adsorbent. Inductively coupled plasma optical emission spectrometer (ICP-OES, Leeman Prodigy 7, Teledyne Leeman Labs., Hudson, NH, USA) with an axial view was used to detect the concentration of metal ions in solution and the employed gas was argon with 99.9999% purity. The operating parameters of ICP-OES for the analytical measurements were: 1.1 Kw RF generator power; 12.0 L/min plasma gas flow rate; 0.5 L/min auxiliary gas flow rate; 34 PSI (pounds per square inch) nebulizer gas flow rate; 16 L/min coolant gas flow rate and 30 s sample uptake delay. The chosen emission lines (nm) included 197.819, 242.795 and 267.595 for Au, 206.200 and 213.856 for Zn, 265.117 and 303.907 for Ge, 324.754 ad 224.700 for Cu, 279.553 and 280.271 for Mg, 249.722 and 249.677 for B. ICP-OES was calibrated with a standard solution of the mixed elements (0, 1, 10 and 20 mg/L).

## 3. Results and Discussion

### 3.1. Characterization

**Figure 1:**
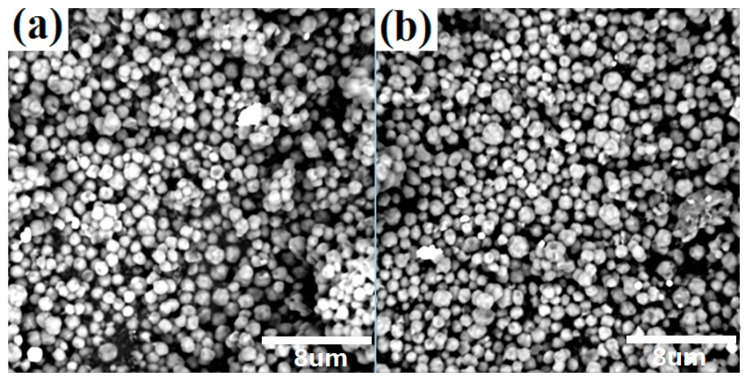
SEM images of PGMA (**a**) and A-PGMA (**b**).

The SEM images of PGMA and A-PGMA were shown in Figure 1. The morphology of the two samples was microspheres. Compared with PGMA, the particle sizes of A-PGMA was almost unchanged. The particle sizes of the two samples were about 1.0 ± 0.2 um.

**Figure 2:**
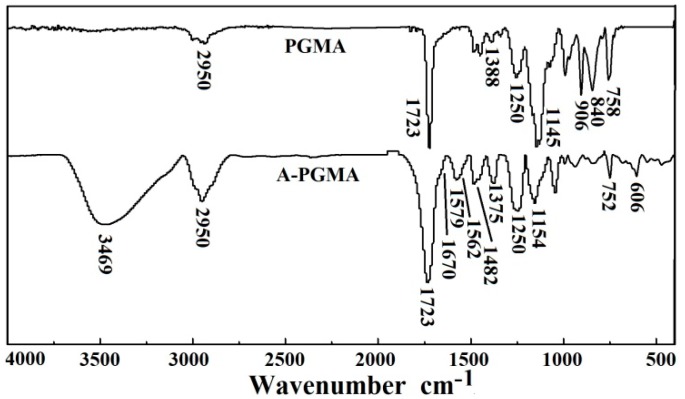
FT-IR spectra of PGMA and A-PGMA.

Figure 2 showed the FT-IR spectra of PGMA and A-PGMA. The peaks at 758 cm^−1^, 1145 cm^−1^, 1250 cm^−1^, 1388 cm^−1^, 1723 cm^−1^ and 2950 cm^−1^ were assigned to C–O–C, C–O, C–C, –CH_3_, C=O and C–H bond, respectively. The epoxide group at 906 cm^−1^ and 840 cm^−1^ can be seen clearly in PGMA and disappeared after ring-opening reaction. After functionalized, new peaks at 606 cm^−1^, 1482 cm^−1^, 1562 cm^−1^, 1579 cm^−1^, 1670 cm^−1^ and 3469 cm^−1^ in A-PGMA were assigned to the bonds of C–S, C–N, C=N, N–H, C=C and O–H. It can be found that the bond at 1732 cm^−1^ was broader because the peaks of C=O stretching and bending vibration overlapped the C=C stretching vibration. Thus, A-PGMA was successfully synthesized.

**Figure 3:**
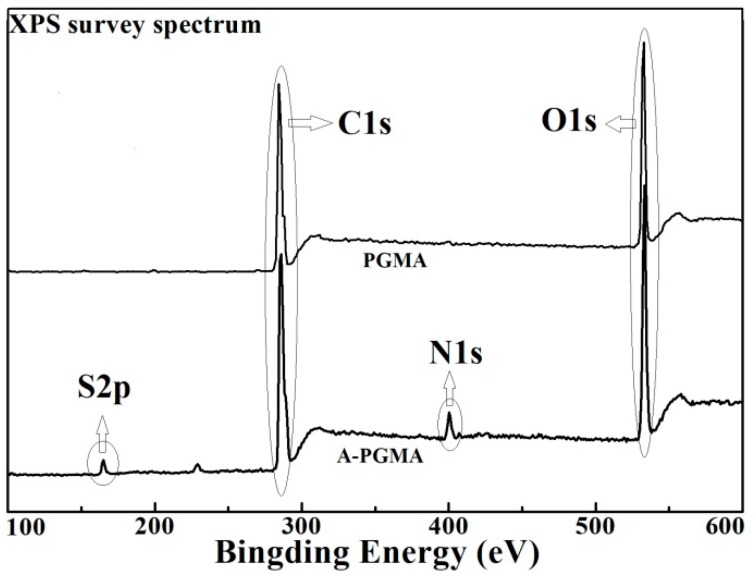
XPS survey scan of PGMA and A-PGMA.

**Figure 4:**
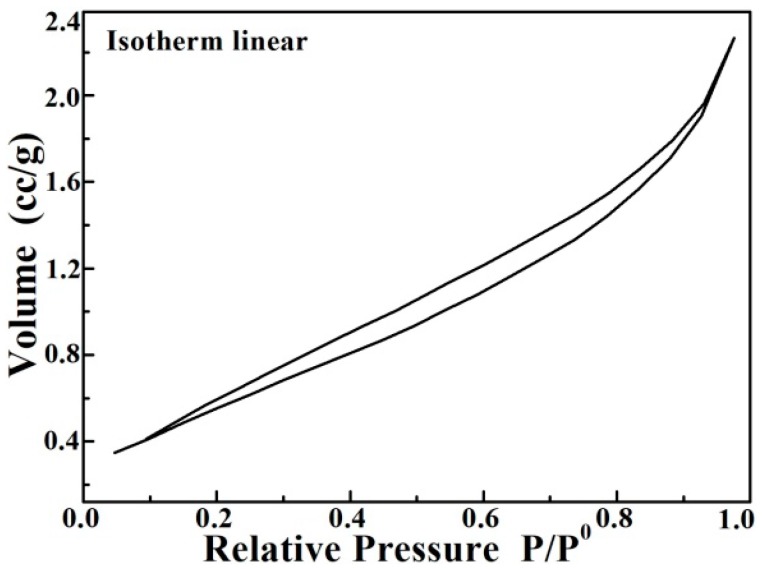
The isotherm linear of BET surface area.

The XPS survey of PGMA and A-PGMA w-as showed in Figure 3. PGMA has only carbon and oxygen. The nitrogen and sulfur atoms were appeared after the ring opening reaction of PGMA. The S2p and N1s spectrum in the XPS survey of A-PGMA further explained that the A-PGMA was successfully synthesized. Figure 4 is the isotherm linear of BET surface area and showed that the specific surface area is 2.26 × 10^4^ cm^2^/g.

### 3.2. Adsorption Behavior of Gold on A-PGMA

#### 3.2.1. Effect of pH on Gold Adsorption

**Figure 5:**
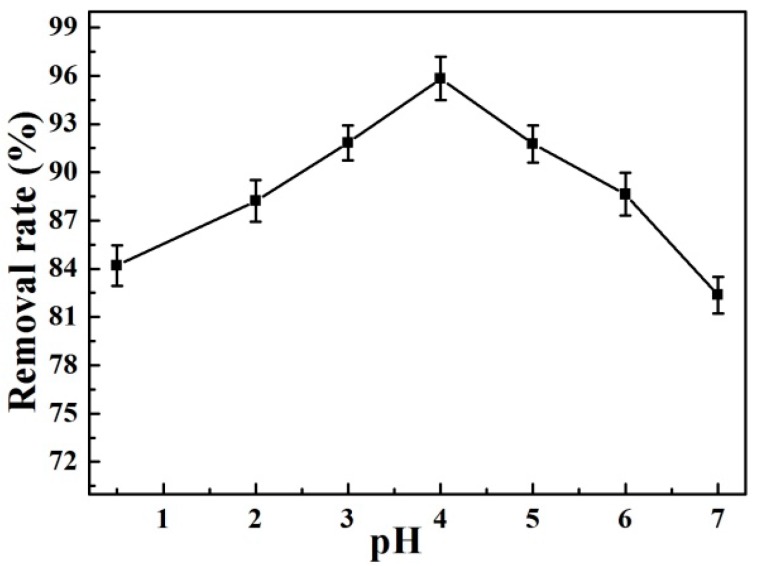
Effect of initial pH on gold adsorption.

**Figure 6:**
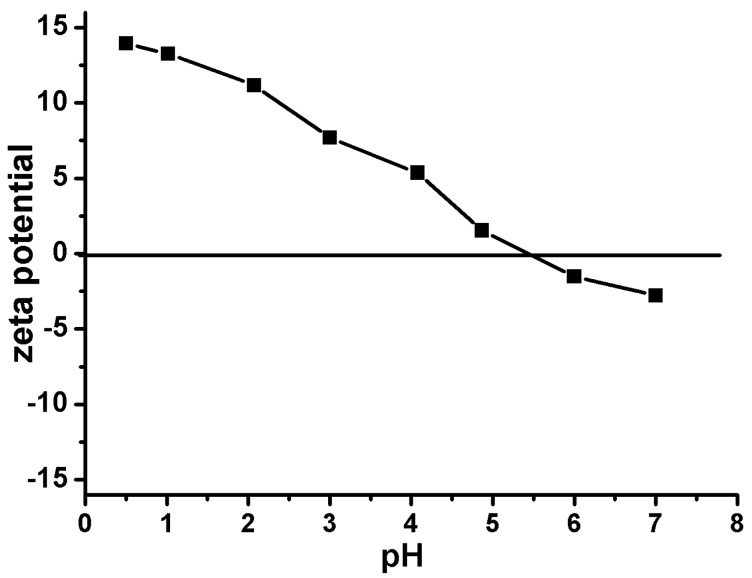
Zeta potential of A-PGMA.

The pH value always plays a key role in the adsorption experiment. The solution acidity affected the adsorption efficiency of metal ions due to the fact that the pH value affected both gold speciation and the surface charge of the absorbents [28,29]. The adsorption of gold ions was researched within the pH range from 0.5 to 7.0 (Figure 5). The adsorption rate increased with the increasing of the solution pH and reached a maximum value at pH 4. The maximum removal rate was 95.8%. The Cl^−^ concentration in the low pH solution was higher and gold ions exist mainly in the form of AuCl_4_^−^. The Cl^−^ ions will compete with chloro-gold ions to seize the adsorption sites; therefore, low adsorption capacities were observed at low pH [30,31]. Figure 6 has shown that the isoelectric point of A-PGMA was about 5.4. The zeta potential of A-PGMA is positive at pH 4. The nitrogen and sulfur atoms on the adsorbent were converted to positive centers through protonation [29,32]. A-PGMA can interact with AuCl_4_^−^ ions by ions exchange. With the increasing of pH value, the hydroxo-containing gold complex such as AuCl_3_(OH)^−^ increased in solution, thus leading to a decrease of the adsorption rate [33]. Related reactions were as follows Equations (3)–(6) [34]:

Au(OH)_4_^−^ + H^+^ + Cl^−^ ⇌ AuCl(OH)_3_^−^ + H_2_O (3)

Au(OH)_3_^−^ + H^+^ + Cl^−^ ⇌ AuCl(OH)_2_^−^ + H_2_O (4)

Au(OH)_2_^−^ + H^+^ + Cl^−^ ⇌ AuCl(OH)^−^ + H_2_O (5)

AuCl_3_(OH)^−^ + H^+^ + Cl^−^ ⇌ AuCl_4_^−^ + H_2_O (6)

#### 3.2.2. Effect of Contact Time and Adsorption Kinetics

**Figure 7:**
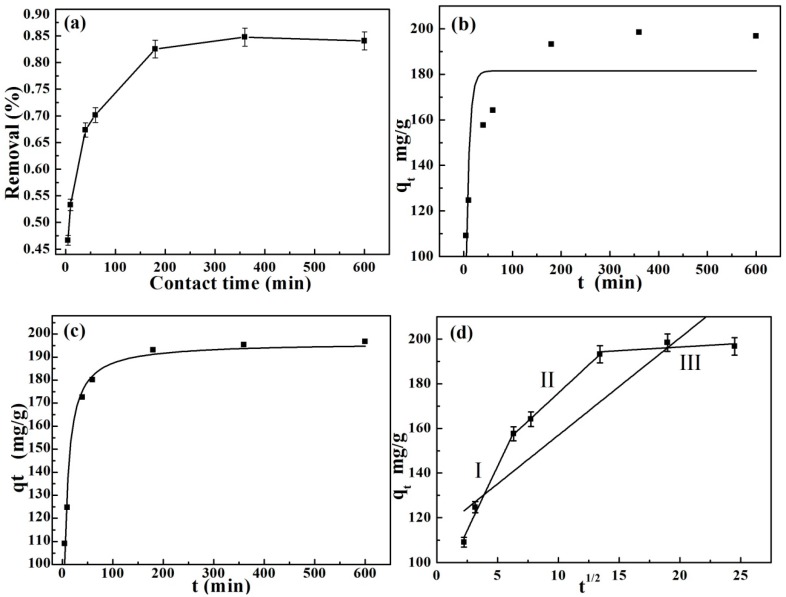
Effect of contact time on the removal rate of gold ions (**a**), the pseudo-first-order (**b**), pseudo-second-order (**c**) and intraparticle diffusion (**d**) adsorption kinetic models of A-PGMA.

The effect of contact time (min) on the removal rate (%) of gold ions was presented in Figure 7a. It can be clearly see that the removal rate of A-PGMA for gold ions increased with the contact time. The removal rate of gold ions increased rapidly within 1 h and finally reached equilibrium at 3 h. The saturated adsorption capacity of gold ions onto A-PGMA was 201.214 mg/g. In the rapid growth stage, gold ions arrive easily to the accessible active sites and bind with the chelating ligands. The diffusion process might be hampered in the equilibrium [32]. With the decreasing of the binding sites, the adsorbent will not continue to adsorb AuCl_4_^−^.

The research of adsorption kinetics is one of the important aspects for the evaluation of the affinity of A-PGMA to gold ions. The pseudo-first/second-order and intraparticle diffusion adsorption kinetic models were used to fit the experimental data, expressing as followed [35,36,37]:

(7) $$\ln(q_{e}-q_{t})=\ln q_{e}-k_{1}t$$

(8) $$\frac{t}{q_{t}}=\frac{1}{k_{2}q_{e}^{2}}+\frac{t}{q_{e}}$$

(9) $$q_{t}=k_{3}t^{0.5}+C$$

where *q_e_* and *q_t_* (mg/g) were the adsorbed capabilities of gold ions at equilibrium time and time (*t*), respectively. *k*_1_ (min^−1^), *k*_2_ (g/(mg·min)) and *k*_3_ (mg/(g·min^0.5^)) referred to the rate constants of the three kinetic models. *C* was the concept about the thickness of boundary layer. 

The nonlinear form of pseudo-first-order and pseudo-second-order models can be expressed by Equations (10) and (11), respectively [38]:

(10) $$q_{t}=q_{e}[1-\exp(-k_{1}t)]$$

(11) $$q_{t}=\frac{q_{e}{}^{2}k_{2}t}{q_{e}k_{2}t+1}$$

The three kinetic models were showed in Figure 7b–d. The correlation coefficients (*R*^2^) and kinetic constants (*k*, *q_e_*) were showed in Table 1. It can be clearly see that the correlation coefficient (*R*^2^) of pseudo-second-order kinetic model (0.9746) was higher than these of pseudo-first-order kinetic model (0.6973) and intraparticle diffusion model (0.7799). The *q_e_* attained by pseudo-second-order kinetic model (196.28 mg/g) was very close to the saturated adsorption capacity (201.21 mg/g). It implied that the pseudo-second-order model was more suitable for the adsorption process of gold ions. It also revealed that the chemical adsorption was the rate-limiting step in the whole gold adsorption process on A-PGMA [39]. Furthermore, the adsorption kinetic model proved that the functional groups on the surface of A-PGMA chelated with gold ions [40]. 

The intraparticle diffusion adsorption kinetic model presented three stages (Figure 7d, Table 2). In stage I (0–40 min), the adsorption capacity increased rapidly due to more available sites on the surface. In stage II (40–180 min), the adsorption rate of gold ions was slower than that of the first stage. It is due to the available sites on the external surface were saturated [37]. In stage III (180–600 min), the adsorption reached equilibrium. In addition, the constants for three stages are not zero. We can see that the intercepting lines of three stages have not gone through the origin. Thus, the intraparticle diffusion model is not the rate-limiting step [41]. 

#### 3.2.3. Effect of Original Gold Concentration and Adsorption Isotherms

**Figure 8:**
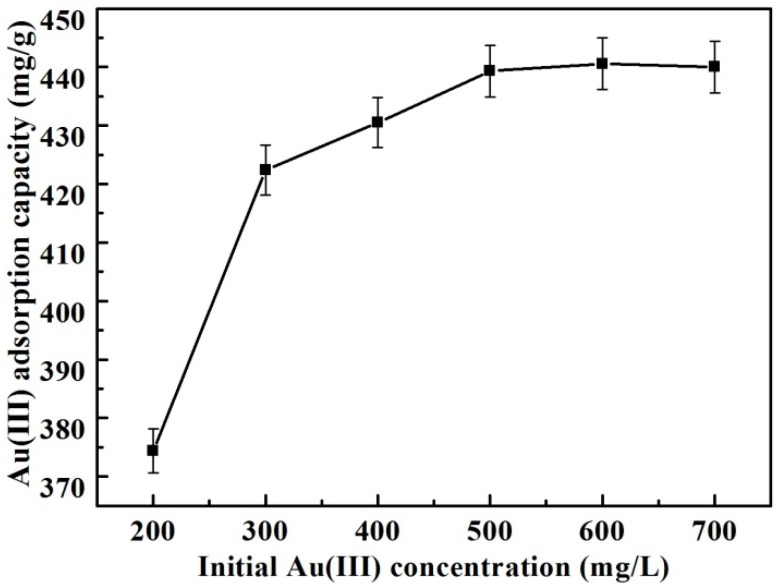
Effect of original gold ions on the adsorption capacity.

Figure 8 has given the influence of original Au(III) concentration on the adsorption capacity. It can be clearly see that the adsorbed amount increased with the increasing of original gold ions concentrations. The Au(III) adsorption capacity increased rapidly within 300 mg/L and gradually become slow, finally reached equilibrium at 500 mg/L. For an original gold ions concentration of 500 mg/L, the Au(III) adsorption capacity was 440.54 mg/g. With the increasing of original gold ions concentration, the adsorbed amount of gold ions had almost no change. This is because the binding sites on the surface of A-PGMA were saturated and less gold ions can be adsorbed on its surface [40]. 

In order to better understand the relationship between adsorption capacity and gold ions concentration, the Langmuir and Freundlich isotherm models were used to analyze the adsorption process of T-PGMA. The Langmuir and Freundlich isotherm models interpreted the monolayer homogeneous and heterogeneous adsorption systems, respectively, could be presented as followed [5,42]:

(12) $$\frac{C_{e}}{q_{e}}=\frac{1}{q_{\max}K_{L}}+\frac{C_{e}}{q_{\max}}$$

(13) $$\ln q_{e}=\ln K_{F}+\frac{1}{n}\ln C_{e}$$

where *q_e_* (mg/g) was the adsorption capacity at equilibrium, *C_e_* (mg/L) and *q*_max_ (mg/g) were the equilibrium concentration and maximum adsorption capacity, respectively. *K_L_* was the constants of the Langmuir model and *K_F_* and n were the constants of Freundlich model. 

The nonlinear form of Langmuir and Freundlich can be expressed by Equations (14) and (15), respectively [43,44]:

(14) $$q_{e}=\frac{q_{\max}K_{L}C_{e}}{K_{L}C_{e}+1}$$

(15) $$q_{e}=K_{F}C_{e}{}^{1/n}$$

**Figure 9:**
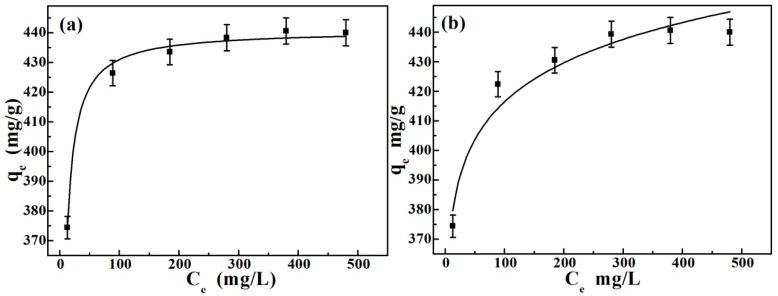
The adsorption isotherms models of A-PGMA.

Figure 9 gave the nonlinear form and Table 3 gave the adsorption parameters of two models, respectively. It can be found that the correlation coefficient (*R*^2^) of Langmuir model (0.9911) was higher than that of the Freundlich isotherm model (0.9389). Simultaneously, the maximum adsorption capacity (*q*_max_) attained by Langmuir model (440.84 mg/g) is very close to the experimental value (440.54 mg/g). In other words, the Langmuir isotherm model described the adsorption of gold ions on A-PGMA very well. The comparison of A-PGMA with other adsorbents were showed in Table 4, and it can be found that the maximum adsorption capacity of A-PGMA (440.54 mg/g) exceeded many adsorbents in literature.

#### 3.2.4. Reusability

**Figure 10:**
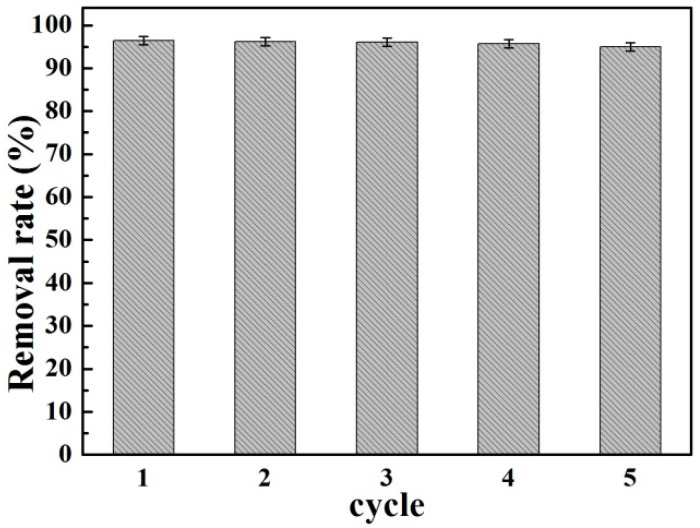
Reusability of A-PGMA.

Reusability is vitally important for an adsorbent. An adsorbent with good reusability can save resources and reduce the cost. To better understand the reusability of A-PGMA, 25 mg A-PGMA was added into 50 mL of the solution with the gold ions concentration of 100 mg/L. After 5 h, the suspension was centrifuged. The supernatant was measured by ICP-OES to obtain the residual concentration of gold ion. The precipitate was desorbed by desorption solution of hydrochloric acid (3 mol/L) and thiourea (1.5 mol/L). After desorption, the precipitate was washed by distilled water five times. Finally, the adsorption and desorption experiments were repeated for four times (Figure 10). After five times of repetitive experiments, the removal rate of gold ions was only decreased from 96.39% to 94.96%. Therefore, A-PGMA has good reusability.

#### 3.2.5. Selectivity

**Figure 11:**
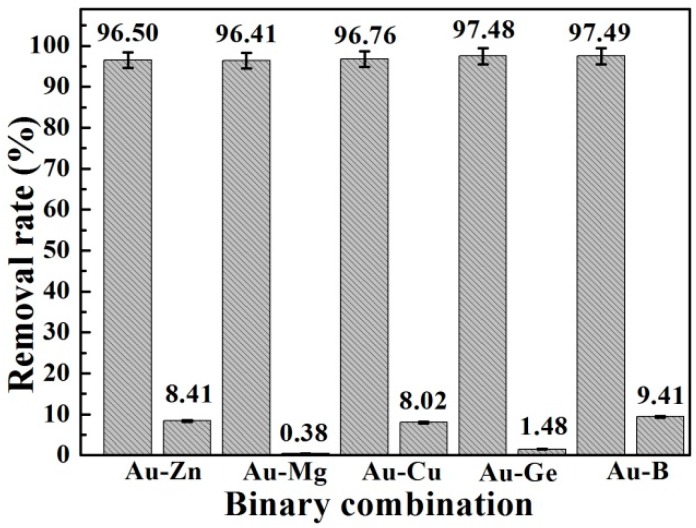
Selectivity of A-PGMA.

A good adsorbent not only has a good reusability, but it also has a good selectivity. In the selectivity adsorption experiment, Zn^2+^, Mg^2+^, Cu^2+^ and Ge^4+^ were selected as coexisting ions (Figure 11). In addition, 10 mg of A-PGMA was added into 20 mL of solutions containing gold ion (100 mg/L) and coexisting ions (100 mg/L), and oscillated it for 5 h at pH 4 and room temperature. After being centrifuged, the concentration of all the coexisting ions was obtained from the supernatant. According to Figure 11, the removal rate of coexisting ions was lower than that of gold ions (96.50%). The results indicated that the A-PGMA chelating resin has a good selectivity for gold ions.

#### 3.2.6. Adsorption Mechanism

**Figure 12:**
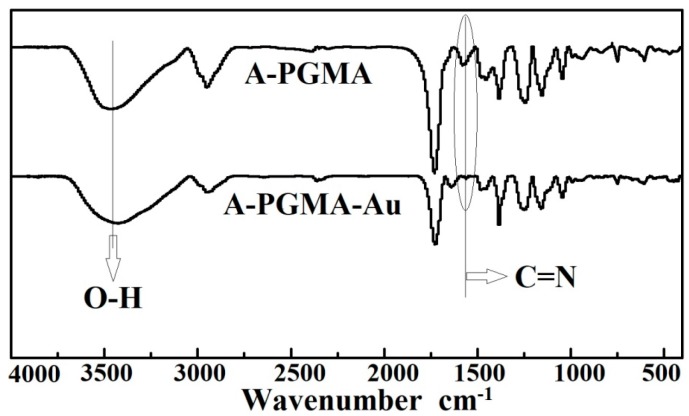
FT-IR spectra of A-PGMA and A-PGMA-Au.

We defined gold ions loaded A-PGMA as A-PGMA-Au. The FT-IR spectra of A-PGMA and A-PGMA-Au were showed in Figure 12. The peak at 1562 cm^−1^ in A-PGMA was attributed to C=N bond. Compared with A-PGMA, it can be found that the C=N bond in A-PGMA-Au was significantly weaker. Therefore, the adsorption was through the chelating binding and ion exchange between Au^3+^ and nitrogen groups on the surface of A-PGMA. 

**Figure 13:**
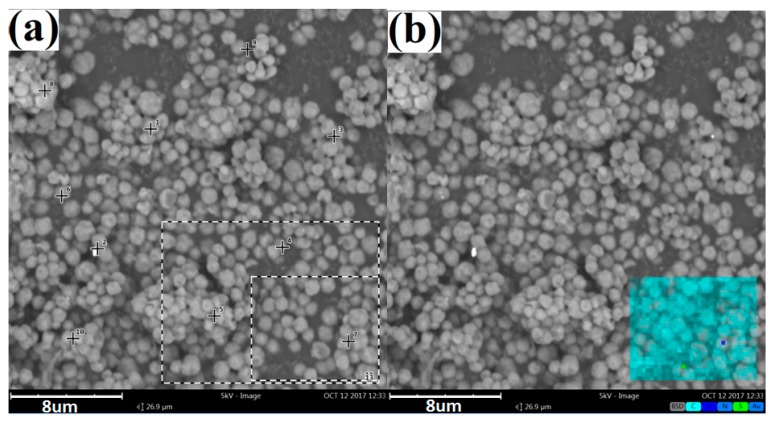

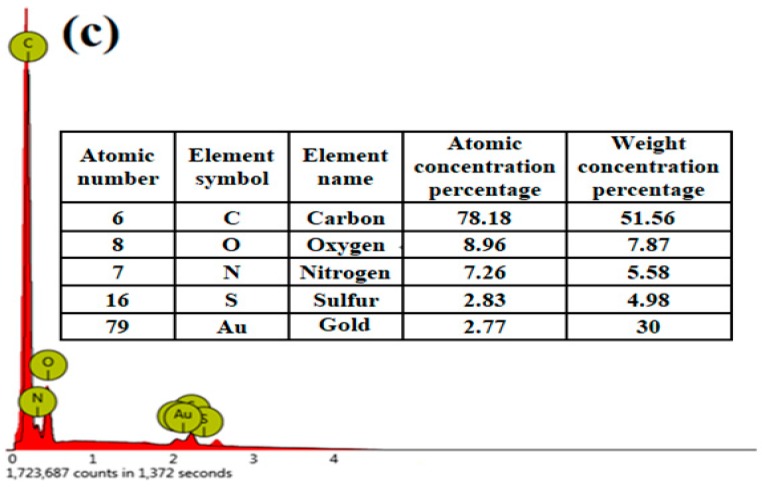
SEM and EDS analysis of A-PGMA-Au.

The SEM and EDS analysis of A-PGMA-Au was showed in Figure 13. Some representative points in Figure 13a were selected for points scanning, part of the surface of A-PGMA-Au was enlarged for surface scanning (Figure 13b) and the analysis results were showed in Figure 13c. The amount of gold ions bound to A-PGMA chelating resin was very high and the results of EDS analysis also indicated that sulfur and nitrogen were the key atoms for gold ion adsorption.

**Figure 14:**
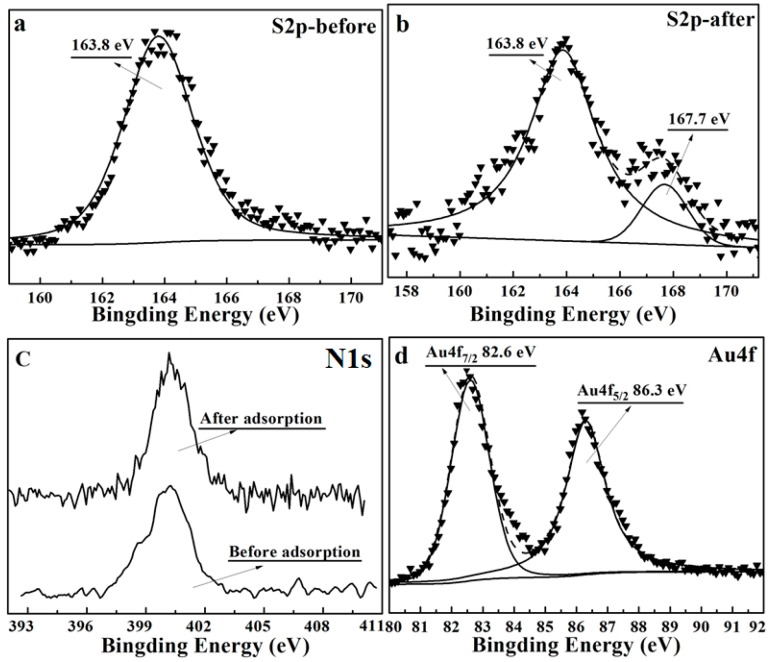
S2p spectra and N1s spectra of before and after gold ions adsorption, and Au4f spectra.

**Scheme 2:**
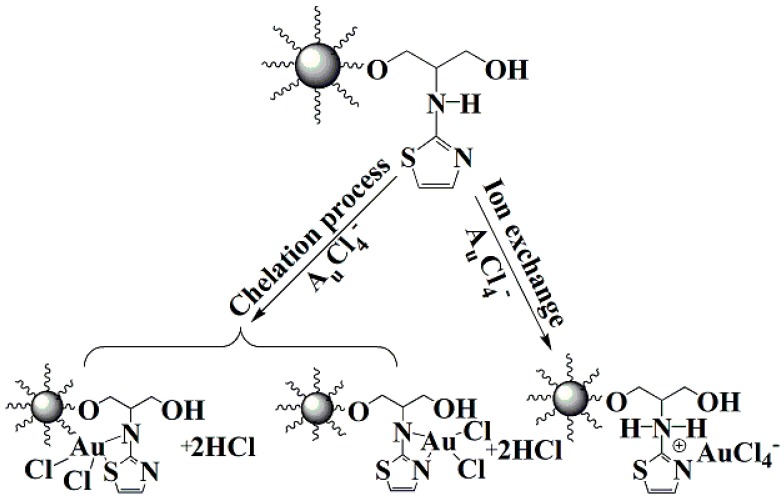
Adsorption mechanism of gold ions on A-PGMA.

In order to better understand the gold ions adsorption mechanism on A-PGMA, the change of S2p, N1sand Au4f spectra were investigated before and after gold ions adsorption (Figure 14). Comparing to the S2p spectra before adsorption, a new peak at 167.7 eV was appeared after gold ions adsorption (Figure 14a,b). The peak at 167.7 was attributed to the chelating of the sulfur atom with the gold ions [29]. The literature has also reported that the sulfur atoms could chelate with AuCl_4_^−^ [18]. After gold ions adsorption, the N1s spectrum shifted from 399.16 eV to 400.30 eV (Figure 14c). The reason can be attributed to the chelating between gold and N atoms in the A-PGMA [54]. It further indicated that the N atoms on the surface of A-PGMA were involved the ion exchange and chelating process. Figure 14d showed that two peaks at 82.6 eV and 86.3 eV were appeared in the Au4f spectra. The binding energies were much lower than that of the free Au(III), indicating that the main adsorption mechanism was ion exchange and chelating [18]. In summary, the adsorption mechanism of gold ions adsorption on A-PGMA were ions exchange and chelation between the sulfur and nitrogen atoms on the surface of A-PGMA and AuCl_4_^−^ (Scheme 2).

## 4. Conclusions

In this study, poly(glycidyl methacrylate) microsphere functionalized with 2-aminothiazole has been successfully synthetized for gold recovery. The new adsorbent was characterized by FT-IR, XPS, BET, Zeta potential and SEM. The maximum adsorption capacity is 440.54 mg/g under optimal pH 4. The adsorption isotherms obeyed well the Langmuir model and the adsorption kinetic was consistent with pseudo-second-order model very well. A-PGMA has a good selectivity from the coexistent ions of Zn(II), Mg(II), Cu(II), and Ge(IV) and a high reusability after five cycles. The adsorption mechanisms of gold ions on A-PGMA were ion exchange and chelation reaction. The A-PGMA has a high potential for gold recovery in industry.

## Figures and Tables

**Table 1:** Kinetics parameters for gold ions adsorption on A-PGMA.

Kinetics Models	Parameters
Pseudo-first-order	*k*_1_ = 0.1458 min^−1^
	*R*^2^ = 0.6973 *q_e_* = 181.47
Pseudo-second-order	*k*_2_ = 0.00107 g/(mg·min)
	*R*^2^ = 0.9746 *q_e_* = 196.28
Intraparticle diffusion	*k*_3_ = 4.3583
	*R*^2^ = 0.7799

**Table 2:** Three stages parameters of intraparticle diffusion kinetics model.

Stage	*k*_3_	*R*^2^	*C*
I	11.4769	0.97895	85.62776
II	5.04205	0.99949	125.4287
III	0.32316	−0.10323	189.948

**Table 3:** Isotherm parameters of gold adsorption.

Isotherms Models	Langmuir			Freundlich		
Parameters	*q*_max_	*K_L_*	*R*^2^	*K_F_*	*n*	*R*^2^
Values	440.84	0.4352	0.9911	338.53	22.237	0.9389

**Table 4:** Comparison of A-PGMA with other adsorbents in literature.

Adsorbents	*q_m_* (mg/g)	Reference
Activated carbon	3.54	[45]
Thiourea–formaldehyde resin	21.56	[46]
Silk sericin and chitosan biosorbents	37.00	[47]
Cellulose	34.40	[48]
Melamine–formaldehyde–thiourea chelating resin	48.00	[49]
Nano-structured Mn_2_O_3_	70.0	[50]
Multiwalled carbon nanotubes	93.5	[51]
Graphene hydrogels	103.50	[52]
Nylon-lycra or PPy	115.00	[53]
A-PGMA	440.54	This work
